# Barriers and facilitators to physical activity uptake and adherence among older South Asians: a qualitative systematic review

**DOI:** 10.1186/s12877-026-07453-3

**Published:** 2026-04-18

**Authors:** Onyinye Ezeokoli, Ivelina Tsocheva, Gurch Randhawa, David Hewson

**Affiliations:** Institute for Health Research, University of Bedfordshire, Bedfordshire, UK

**Keywords:** Cultural influences, Social support, Health beliefs, Ageing and frailty, Exercise participation, Ethnic minority health

## Abstract

**Background:**

There are high levels of physical inactivity and chronic diseases among older South Asians. Although physical activity (PA) offers substantial health benefits for older adults, engagement and maintenance are low in this population. There are reviews of previous studies on South Asians in general, but very few concentrate on older South Asians and the synthesis of qualitative research to inform intervention design for this group of people.

**Objective:**

To comprehensively evaluate the current body of qualitative research on the facilitators and inhibitors of participation in physical activity in older South Asians.

**Methods:**

This qualitative systematic review was reported in accordance with PRISMA 2020. Six major databases were searched between the inception of the databases and April 2025. The inclusion criteria for this systematic review included English-language primary qualitative studies and mixed-methods studies with extractable qualitative findings relating to South Asians aged 65 years and older. The Joanna Briggs Institute tool was used in addressing issues of quality in qualitative research studies. Deductive synthesis of studies used Theoretical Domains Framework, which was further transformed into Capability, Opportunity, Motivation–Behaviour (COM-B) model.

**Results:**

A total of sixteen studies in six countries satisfied the inclusion criteria. Barriers to physical activity (PA) included the participant's perception of physical restrictions, other family care commitments, a lack of knowledge about physical activity, social norms, and low levels of motivation. Facilitators include social support, participation in supervised group activities, health beliefs, facilitating environments, and professional health advice. All can be related mainly to the domains: physical and psychological capabilities, social and physical opportunities, and reflective motivation in the COM-B model.

**Conclusion:**

Engaging in physical activity among the elderly South Asian population is affected by a complex mixture of individual, social, and cultural factors. For South Asians, it is important that interventions be implemented in a manner that meets the needs regarding capability motivation, and support.

**Supplementary Information:**

The online version contains supplementary material available at 10.1186/s12877-026-07453-3.

## Introduction

Physical inactivity is a major modifiable risk factor for non-communicable disease, functional decline, and loss of independence in later life. In older adults, regular physical activity is associated with improved physical function, mental wellbeing, maintenance of independence, and reduced risk of chronic disease [1]. Despite these benefits, many older adults do not achieve recommended levels of physical activity, and barriers in later life may differ from those seen in younger populations [2]. Physical activity participation tends to decline with age, and lower levels of participation have been reported in some minority ethnic populations. Despite these benefits, many older adults do not achieve recommended levels of physical activity, and barriers differ in older age [3, 4]. This pattern is especially concerning given the disproportionate burden of cardiometabolic disease and mobility-related health risks reported in some South Asian communities [5, 6].

In the South Asian older population, the prevalence of cardiometabolic conditions such as type 2 diabetes and cardiovascular diseases, as well as musculoskeletal conditions due to a sedentary lifestyle, is abnormally high. There is considerable evidence suggesting a lower adherence rate to recommended levels of physical activity among South Asians than among Caucasians of White European descent, with increasingly wider gaps during old age [7, 8].

The reasons older South Asians are or are not participating in physical activity thus have important implications in the reduction of inequities in health. While quantitative methodologies have identified a low level of physical activity, plus the subsequent risks to physical and mental health, in this demographic, these are not very informative in relation to the contextual aspects surrounding the behaviour. The role of qualitative research is more informative in relation to beliefs, attitudes, social norms, and those aspects within environments surrounding physical activity, especially among older populations where physical, family, and social factors meet.

There have been several qualitative, mixed, or other forms of research that have examined barriers and facilitators for PA for South Asian adults as well as the general older adult population. Many previous systematic reviews have noted the existence of various influencing factors, such as those due to the environment (e.g. weather, security, and facility availability), communication/social issues (e.g. language barriers, poor health literacy, lack of social support), as well as those related to culture or beliefs (e.g. fatalism, inappropriateness of exercise in their culture) [8–11]. Although useful, most are for South Asian adults irrespective of age.

Older adulthood is also commonly associated with multimorbidity, loss of physiological function, and impaired functionality, making individuals even more susceptible to frailty. Frailty can be viewed as either a biological phenotype with symptoms and manifestations of weakness, fatigue, and decreased physical activity [12] or as an additive effect of various health deficits such as diseases, symptoms, and disability [13]. Such biological changes could pose additional barriers to PA participation,however, there is a paucity of qualitative literature encompassing viewpoints held by older South Asians with functional and health limitations.

As of present, there is no known qualitative systematic review focusing on synthesizing evidence on barriers and facilitators towards engagement and adherence with PA in older South Asians. When primary literature is limited regarding direct assessments of frailty, a qualitative synthesis on studies involving older South Asians affords a significant opportunity towards discerning factors related to individuals at risk of frailty, including health-related considerations, personal perceptions of physical impairment, and social supports.

Previous reviews have examined barriers and facilitators to physical activity among South Asian adults more broadly, or among ethnically diverse older adults. However, these approaches may obscure determinants that are particularly salient in later life, such as multimorbidity, functional limitations, changing family roles, and dependence on culturally acceptable social opportunities for activity. To our knowledge, no qualitative systematic review has specifically synthesised evidence on barriers and facilitators to physical activity uptake and adherence among older South Asians. This review addresses that gap by focusing on older South Asians as a distinct subgroup and by mapping qualitative findings to the Theoretical Domains Framework (TDF) and the COM-B model to generate theory-informed implications for intervention design.

Furthermore, very few existing reviews have utilised a theory-driven approach to integrate qualitative findings into behavioural processes. This is particularly important because there is now evidence that using such frameworks leads to improved intervention design, ensuring key domains or constructs are not omitted. The COM-B model [14], and TDF [15], together, offer a comprehensive, structural approach to understanding health behaviour, and evidence translation to facilitate intervention design. This approach has been supported in terms of its contributions to complex intervention design.

To address the identified knowledge gap, the current qualitative systematic review will integrate qualitative data on the facilitators and barriers to PA uptake and adherence in older South Asians to yield theoretically informed findings that can be utilized to inform the design of culturally appropriate and age-appropriate PA interventions by application of the COM-B and TDF frameworks.

### Aim

To synthesise qualitative evidence on barriers and facilitators to physical activity uptake and adherence among older South Asians.

### Objectives

1. To identify barriers and facilitators to physical activity uptake and adherence reported by older South Asians.
2. To map these findings to the TDF and COM-B model.
3. To derive implications for culturally and age-appropriate physical activity interventions.

### Design and reporting standards

This systematic qualitative review and reporting have been carried out in accordance to the PRISMA 2020 guidelines [16]. The qualitative systematic review approach has been used to inform the review process to gain insight into the experiential and cultural factors that stimulate physical activity in older South Asian communities.

### Eligibility criteria

Studies were eligible if they: (1) reported qualitative findings relating to South Asian adults aged 65 years and older; (2) explored barriers and/or facilitators to physical activity uptake, participation, or adherence; (3) were primary qualitative studies or mixed-methods studies with extractable qualitative findings; and (4) were published in English.

Studies with broader age ranges or mixed ethnic samples were included only where findings relating specifically to South Asian participants aged ≥ 65 years were explicitly reported in the text, quotations, tables, or subgroup analyses, or could be clearly isolated following full-text review. No data from participants aged < 65 years were synthesised.

Exclusion criteria were quantitative-only studies, studies without extractable qualitative findings, studies in which age- or ethnicity-specific data for South Asians aged ≥ 65 years could not be isolated, studies not addressing physical activity behaviour, conference abstracts, and non-English publications.

### Information sources and search strategy

A systematic search was conducted in MEDLINE, Web of Science Core Collection, CINAHL, SPORTDiscus, APA PsycInfo, and Allied and Complementary Medicine Database between database inception and April 2025. These databases were selected to ensure coverage across medical, public health, behavioural science, nursing, and allied health literature relevant to physical activity and qualitative research.

The search strategy combined controlled vocabulary terms and free-text keywords relating to: (1) South Asian ethnicity; (2) physical activity/exercise; and (3) qualitative inquiry and barriers/facilitators to participation. Search terms were adapted for each database. To improve search sensitivity, potentially relevant studies were not excluded at the search stage solely because barriers or facilitators were not explicitly stated in the title or abstract. In addition to database searching, backward citation searching of included studies and relevant reviews, and hand-searching of reference lists, were undertaken. The full search strategy for MEDLINE, together with adapted strategies for the other databases, is provided in Supplementary File 1. Search results from all databases were exported to EndNote 21 for de-duplication prior to screening. The search strategy was developed using a structured PICO framework and included combinations of terms relating to South Asian ethnicity, physical activity, and behavioural determinants. Detailed search strings, including Boolean operators and database-specific syntax, are also found in Supplementary file 1.

To maximise sensitivity, studies were not excluded solely because barriers or facilitators were not explicitly mentioned in the title or abstract. Potentially relevant studies were assessed during the screening stage to minimise the risk of omitting relevant qualitative evidence.

The full database search strategy is provided in Supplementary File 1. Search strategies were adapted for each database according to database-specific indexing terms and syntax.

### Study selection

All retrieved records were imported into EndNote 21, and duplicate records were removed. Title and abstract screening was conducted to identify potentially eligible studies. The full text articles were screened for eligibility based on predetermined inclusion criteria from retained research articles. Screening and selection for study eligibility were independently conducted by the two authors with discrepancies resolved by mutual discussion. Where eligibility could not be determined due unclear reporting (e.g. age and ethnicity of study participants), corresponding authors were approached for further clarification and information. Full-text articles of retained records were then assessed against the inclusion criteria. The study selection process is presented using a PRISMA flow diagram.

### Quality appraisal

The methodological quality of these studies was critically assessed using the Joanna Briggs Institute (JBI) Critical Appraisal Checklist for Qualitative Research [17]. The tool evaluates the integration of research method and philosophical underpinning, data collection and analysis approach, results interpretation and voice representation of participants, as well as consideration of researcher and ethical approvals.

Each study was appraised independently, and overall quality ratings were used to support interpretation of findings rather than to exclude studies.

### Data extraction

A structured Microsoft Excel sheet was used for data extraction. Extracted information included author and year, country and setting, participant characteristics (ethnicity, age, gender, and migration status), study design, data collection method, and qualitative findings relating to barriers and facilitators to physical activity. For studies involving broader age ranges or mixed ethnic samples, extraction was restricted to quotations, author interpretations, and subgroup findings explicitly attributable to South Asian participants aged ≥ 65 years. Where this could not be established confidently, the study was excluded from synthesis. Relevant participant quotations were extracted where available to support transparency of interpretation.

### Data synthesis

Qualitative findings were synthesised using a directed content analysis approach informed by the Theoretical Domains Framework (TDF). Extracted participant quotations and author interpretations were coded to relevant TDF domains and constructs. Multiple coding was permitted where a finding related to more than one domain. The coded domains were then mapped to the corresponding COM-B components (physical capability, psychological capability, physical opportunity, social opportunity, reflective motivation, and automatic motivation) using established links between the TDF and COM-B model.

Coding and framework mapping were undertaken by the review team. Any uncertainties or disagreements regarding coding were resolved through discussion among the authors until consensus was reached. Although the synthesis was primarily deductive, interpretation remained iterative, and attention was given to contextual nuances within the included studies in order to reduce the risk of over-constraining findings within predefined behavioural domains. All extracted findings were independently reviewed by a second reviewer to enhance consistency.

### Registration protocol (PROSPERO statement)

This review was not registered in PROSPERO. We acknowledge that PROSPERO can accept systematic reviews of qualitative evidence under appropriate circumstances. However, the review question, eligibility criteria, search strategy, and analytic approach were specified a priori and are reported transparently in the manuscript and supplementary materials.

## Results

### Study selection

**Fig. 1:**
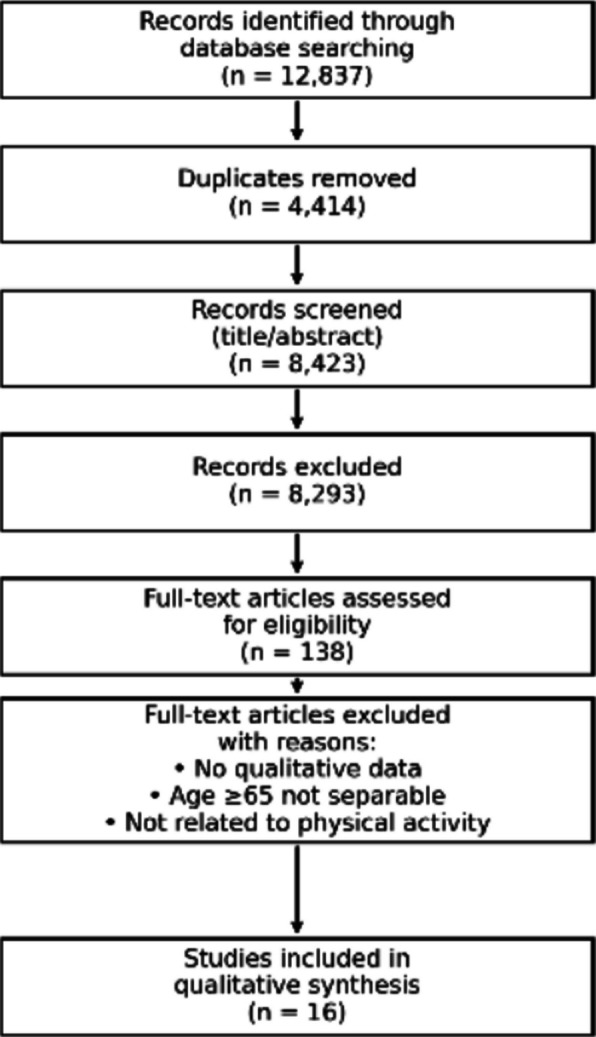
PRISMA 2020 flow diagram of study selection

The initial search of the databases yielded 12,837 records, from which 4,414 were removed due to the presence of duplicates. Subsequently, 8,293 were removed based on the title and abstract. A total of 138 full-text articles were screened, including eight additional records retrieved through hand searching. The final pool of 16 observational studies met the criteria for inclusion in the systematic review. Reasons for exclusion at the full-text screening stage included lack of extractable qualitative data, inability to isolate findings specific to South Asian participants aged ≥ 65 years, and lack of relevance to physical activity behaviour. The study selection process is shown in the PRISMA flow diagram (Fig. 1).

### Study characteristics

The 16 research studies included in this analysis were published between 2004 and 2023. They were carried out in six different countries: United Kingdom (*n* = 7), Canada (*n* = 4), United States (*n* = 2), India (*n* = 1), Pakistan (*n* = 1), and Australia (*n* = 1). The research participants consisted of members from Indian, Pakistani, Bangladeshi, Punjabi Sikh, Nepalese, and Mixed South Asian communities. In seven research studies, migration status was identified. Migration was primarily among first-generation migrants.

Most included studies were conducted in high-income countries and involved migrant South Asian populations, particularly first-generation migrants. Only two studies were conducted in South Asia itself. This should be considered when interpreting transferability of the findings.

Of the 16 included studies, several involved broader age ranges and/or mixed ethnic samples. In these cases, only findings explicitly attributable to South Asian participants aged 65 years and older were extracted and synthesised.

### Inclusion criteria

Included studies comprised primary qualitative studies and mixed-methods studies with extractable qualitative findings. Although several studies included broader age ranges and/or mixed ethnic samples, only findings explicitly relating to South Asian participants aged 65 years and older were extracted and synthesised in this review.

Characteristics of studies have been summarised in Table 1 below.

**Table 1:** Characteristics of included studies

Authors (Year)	Country	City	Ethnicity of all participants	Migration status	Number of participants (original study)	Age range of original study sample	Age group analysed in this review	Study design	Data collection
Asamane et al. [18]	UK	Birmingham	African, Indian, Pakistani, Bangladeshi or Caribbean	NA	20 Pakistanis (3F); 6 Indians (1F); 4 Bangladeshis (0F)	60 +	** ≥ 65 years**	Qualitative	Semi-structured interviews
Caperchione et al. [19]	Canada	Calgary, Alberta	South Asians (Punjabi), others	NA	41 Indians; 2 Pakistanis; others	18 +	** ≥ 65 years**	Mixed methods	CATI
Castaneda-Gameros et al. [20]	UK	NA	South Asians, African/Caribbean, Arab, White Irish	First generation	60 females	Mean 70.8 (SD 8.1)	** ≥ 65 years**	Mixed methods	Semi-structured interviews
Cross-Bardell et al. [21]	UK	East Midlands	Pakistani, Indian, Bangladeshi	NA	34	19–67	** ≥ 65 years**	Qualitative	Semi-structured interviews
Dave et al. [22]	USA	Chicago, IL	South Asian women	NA	42	18–71	** ≥ 65 years**	Qualitative	Focus groups
Fernandes et al. [23]	Australia	Melbourne	Indian	First & second generation	21	18–65	** ≥ 65 years**	Qualitative	Semi-structured interviews
Gour et al. [24]	India	Ujjain	Indian	NA	30–36	65–70	** ≥ 65 years**	Qualitative	Focus groups
Horne et al. (2012b) [25]	UK	Northwest England	South Asians	NA	46	60–70	** ≥ 65 years**	Qualitative	Interviews & FGDs
Horne et al. [26]	UK	NA	Indian, Pakistani, White British	First generation	127	60 +	** ≥ 65 years**	Qualitative	Interviews & FGDs
Kalavar et al. [27]	USA	NA	Asian Indian	First generation	10	66–79	** ≥ 65 years**	Qualitative	Focus groups
Mahmood et al. [28]	Canada	Metro Vancouver	South Asians	First generation	22	40–79	** ≥ 65 years**	Qualitative	Focus groups
Oliffe et al. [29]	Canada	British Columbia	Punjabi-Sikh men	First generation	36	50–83	** ≥ 65 years**	Qualitative	Observation & interviews
Pardhan et al. [30]	UK	Peterborough & Cambridge	Pakistani, Nepalese, Indian	NA	35	30 +	** ≥ 65 years**	Qualitative	Focus groups
Salma et al. [31]	Canada	Edmonton	South Asians, Arab, African	First generation	68	< 75	** ≥ 65 years**	Qualitative	Interviews & FGDs
Tariq et al. [32]	Pakistan	Lahore	Pakistani	NA	47	30–70 (PLwD); 18 + (family)	** ≥ 65 years**	Qualitative	Semi-structured interviews
Victor [33]	UK	NA	Pakistani, Bangladeshi	NA	109	50 +	** ≥ 65 years**	Qualitative	Interviews

*Abbreviations*: *F* female, *NA* not available, *FGD* focus group discussion, *CATI* computer-assisted telephone interview, *PLwD* people living with diabetes

Although some included studies recruited participants younger than 65 years, only qualitative findings explicitly relating to participants aged ≥ 65 years were extracted and included in the synthesis. No data from participants aged < 65 years contributed to the results of this review

Although several included studies involved wider age ranges, only qualitative findings relating to participants aged 65 years and older were extracted and synthesised in this review.

### Methodological quality

Quality appraisal was done using the Joanna Briggs Institute's Critical Appraisal Checklist for qualitative studies. The overall methodology quality for the included studies was found to vary from moderate to high. Most studies were judged to be of moderate to high methodological quality. The study quality is presented in more detail in Table 2.

**Table 2:** JBI critical appraisal of included articles

Authors (Year)/JBI critical appraisal checklist	Appraisal checklist 1	Appraisal checklist 2	Appraisal checklist 3	Appraisal checklist 4	Appraisal checklist 5	Appraisal checklist 6	Appraisal checklist 7	Appraisal checklist 8	Appraisal checklist 9	Appraisal checklist 10	Total	Overall Appraisal
Asamane et al. [18]	Yes	Yes	Yes	Yes	Yes	Yes	Yes	Yes	Yes	Yes	10	Include
Caperchione et al. [19]	No	Yes	Yes	Yes	Yes	Yes	Yes	Yes	Yes	Yes	9	Include
Castaneda-Gameros, Redwood, and Thompson [20]	No	Yes	Yes	Yes	Yes	No	No	Yes	Yes	Yes	7	Include
Cross-Bardell et al. [21]	No	Yes	Yes	Yes	Yes	No	Yes	Yes	Yes	No	7	Include
Dave et al. [22]	No	Yes	Yes	Yes	Yes	No	Yes	Yes	No	Yes	7	Include
Fernandes, Hinckson, and Richards (2023) [23]	No	Yes	Yes	Yes	Yes	Yes	Yes	Yes	Yes	Yes	9	Include
Gour et al. [24]	No	Yes	Yes	Yes	Yes	No	Yes	No	Yes	Yes	7	Include
Horne et al. (2012b) [25]	No	Yes	Yes	Yes	Yes	No	Yes	Yes	Yes	Yes	8	Include
Horne et al. [26]	No	Yes	Yes	Yes	Yes	No	Yes	Yes	Yes	Yes	8	Include
Kalavar et al. [27]	No	Yes	Yes	Yes	Yes	No	Yes	No	Yes	Yes	7	Include
Mahmood et al. [28]	Yes	Yes	Yes	Yes	Yes	No	Yes	Yes	Yes	Yes	9	Include
Oliffe et al. [29]	No	Yes	Yes	Yes	Yes	No	Yes	Yes	Yes	No	7	Include
Pardhan et al. [30]	No	Yes	Yes	Yes	Yes	No	No	No	Yes	Yes	6	Include
Salma et al. [31]	No	Yes	Yes	Yes	Yes	Yes	Yes	Yes	Yes	Yes	9	Include
Tariq, Rosten, and Huber [32]	No	Yes	Yes	Yes	Yes	No	No	Yes	Yes	Yes	7	Include
[33]	No	Yes	Yes	Yes	Yes	Yes	Yes	No	Yes	Yes	8	Include

Across the included studies, reporting was generally strongest in relation to alignment between research aims, qualitative methodology, data collection, and interpretation. Participant voice was usually represented adequately through quotations or author interpretation. However, several studies provided limited information on philosophical positioning, reflexivity, and the influence of the researcher on the research process. These limitations were taken into account when interpreting the strength and transferability of findings, but studies were not excluded on appraisal grounds alone because they still contributed relevant contextual insights to the synthesis.

### Construction of barriers and facilitators of physical activity

The results were coded and interpreted using TDF-COM-B. Barriers and facilitators of engagement and adherence with physical activity were identified for all six components of COM-B. The key findings are described below, and illustrative quotes are included in Table 3.

**Table 3:** Identified COM-B domain(s)and their associated TDF domains and constructs

Article (Year)	Country	Ethnicity	COM-B Domain(s)	TDF Domain(s)	TDF construct(s) and subthemes	Key Results	Examples of quotes
Gour et al. [24]	India	Indians	Automatic motivation; Physical opportunity; and Reflective motivation	Emotion; Environmental context and resources; and Goals	**Positive affect; Resources; and Implementation intention** (Engagement in PA is promoted by it’s been enjoyable, it been supervised, and a desire to improve one's health)	The perceived health benefits and enjoyment of supervised PA make it more likely for people to engage in it	“Initially, I thought I might get hurt while doing yoga or exercise as the body grows weak with age. Carelessness is, in my opinion, the biggest reason. I was practicing in my home in the wrong way, with negative impact or no impact. We can’t practice at home like a class. I try it at home, but it wasn’t as enjoyable as it was in the classroom. We have to be correct in the classroom; we can do what we want at home. Therefore, I continued to enjoy the class and get more health benefits.” During intervention, male participant
Castaneda-Gameros, Redwood, and Thompson [20]	UK	Pakistanis, Indians, and Bangladeshis	Physical capability	Physical skills	**Ability** (perceived physical limitation discourages participation in PA)	Physical limitation are barriers to PA participation	“The doctor's saying [to walk] but if I can’t do it even if the doctor's saying, how can I do it? (Bangladeshi, 81y, frail)”
Horne et al. [26]	UK	Indians and Pakistanis	Physical opportunity	Environmental context and resources	**Environmental stressors** (Time constraints because of family commitments deters PA engagement)	Time constraints is a potential barrier to PA engagement	Participant 4 (SA male, 68yrs: Less active): Yeah, I have a lot of family responsibilities and to look after my wife and everything... no time for exercise
Horne et al. [26]	UK	Indians and Pakistanis	Physical opportunity; and psychological Capability	Environmental context and resources; and Knowledge	**Environmental stressors: and Knowledge** (Time constraints and lack of PA knowledge deters PA engagement)	Time constraints and not being knowledgeable about PA are potential barriers to PA engagement	Participant 7 (SA male, 70yrs: Active):... these people are mainly from farming clan in India and there they have a lot to do such as bending, carrying, cropping etc. It is the environment and the culture that you don’t really have time or know about exercise. There is too much to do
Oliffe et al. [29]	Canada	Indians	Physical opportunity; and social opportunity	Environmental context and resources; and social influences	**Resources: and social support** (Available walking track and social interaction promote PA participation)	Available walking track and social interaction promote PA	I walk, there is a track here, and the other thing is the friends that I have made here. I chit chat with them and pass my time quite nicely. Then by the time I get home, the children are home from work, and then I mingle with them. (67-year-old man)
Gour et al. [24]	India	Indians	Physical opportunity; Physical capability; and social opportunity	Environmental context and resources; Physical skills; and social influences	**Environmental stressors; Ability; Social support** Household commitments, perceived physical limitation, and lack of family support are potential barriers to PA engagement	Household commitments, perceived physical limitation, and lack of family support are potential barriers to PA engagement	“I have never had time; I am still occupied with homework. I could never practice either yoga or exercise because of too much stress, even though I wanted to. I’m taking care of my grandson. I can’t abandon him and come, so he doesn’t go anywhere. I’m overweight and I can’t do any sort of exercise. Even if I join your program, I will not be able to continue! I have a lot of questions about getting my family support.” Before intervention, female participant
[33]	UK	Pakistanis, and Bangladeshis	Psychological capability	Behavioural regulation	**Self-monitoring/Breaking habit** Unwillingness to engage in PA	Dislike of PA is a potential barrier	Zakira, 65, commented that she did not like to exercise although she had been advised to do so by her doctor “he said to do exercise and walk but I do not like it very much.”
Gour et al. [24]	India	Indians	Psychological Capability; and Physical opportunity	Social influences; and Environmental context and resources	**Knowledge: and Resources** (Health practitioner advice and free PA sessions are motivation to PA engagement)	Health practitioner advice and free sessions are a motivation to be PA	“I went to the Orthopedic, he advised me to join the yoga/light exercise class, he told me that medicines would not help. Usually we’re going to have to pay yoga/light exercise class, but I’m going to get the class free of charge.” During intervention, female participant
Horne et al. (2012b) [25]	UK	Indians and Pakistanis	Reflective motivation	Goals	**Implementation intention** (Engagement in PA is promoted by a desire to improve one's health and maintain independence)	PA is perceived to improve one's health to remain independent	South Asian male, 70 years; active: I like to be fit. I don’t want to need anybody’s help… I like to do everything myself if I can to stay independent…
Asamane et al. [18]	UK	Pakistanis, Indians, and Bangladeshis	Social opportunity	Social influences	**Social support** (PA with social and group component encourages participation)	Group activity can encourage PA participation, improves social networks and physical function	“... So, when I joined here, it has help[ed] me [to] socialise, and the group exercise is keeping me active, my [physical] function is improving” (P15, 75 years, Pakistani, Male)
Gour et al. [24]	India	Indians	Social opportunity; and Reflective motivation	Social influences; and Goals	**Social support: and Implementation intention** (Social support and the desire to improve one's health encourages participation in PA)	Social support and perceived benefits make it more likely for people to engage in PA	“The instructors pay attention to each participant and provide handhold support. My mind is very peaceful right now. I used to worry a lot earlier. In our lives, we had hopelessness, disinterest. But all negative emotions were gone. We used to get tired easily, and that fatigue haunted us mentally, I used to feel old, and my time is almost over, after yoga/light exercise, I feel like I’m young.” After intervention, male participant

Details: *UK* United Kingdom, *NA* Not available, *SA* South Asian, *PA* Physical Activity

### Physical ability

Feelings of physical limitation, pain, illness, or the loss of function that comes with age were identified as common deterrents to physical activities. The loss of strength, feelings of tiredness, or concerns about injuries were identified as personal deterrents to participation in physical activities. Such deterrents were more common among the elderly who suffered from chronic conditions.

Poor knowledge and understanding about physical activity forms appropriate for old age were found as a barrier. Some people felt home chores alone could constitute exercise, and there was lack of knowledge about intensity and type. Beliefs about safety and appropriateness related to physical activity among old people acted as yet another barrier.

### Physical opportunity

The physical activity behaviour was influenced substantially by environmental factors. Barriers included lack of availability of suitable and acceptable facilities; cost; weather; and presence of household responsibilities. Intrinsic factors included the presence of a safe route; free and low-cost options; and supervised opportunities.

### Social opportunity

Social factors emerged as important predictors of physical activity participation. Social influences were an important determinant of physical activity behaviour. Family responsibilities, cultural expectations, and community norms were reported to influence participation in exercise [31, 33]. In particular, some women described feeling discouraged from exercising in public spaces due to concerns about social judgement. As one participant stated: “What would the neighbours think if I was walking in the street?” [33].A lack of familial support, caring responsibilities, and social or cultural pressures—more evident among women—were identified as common deterrents. However, social activities, socializing, and the encouragement of peers and family members were important facilitators.

### Reflective motivation

Beliefs about the benefits and value of physical activity affected participation. Motivation to engage in physical activity was often linked to perceived health benefits and the desire to maintain independence in later life [8, 26]. Participants reported that maintaining mobility and avoiding dependence on others were important reasons for staying active. One participant described this motivation as wanting to remain independent and able to perform daily tasks without assistance. Motivators included personalized beliefs to maintain independence or optimize health benefits or prevent loss of function or enhance mental well-being. When beliefs about the benefit or meaning of the activity were present, participants were more likely to begin or maintain participation.

### Automatic motivation

The behaviours associated with emotional experiences related to physical activity, such as enjoyment, satisfaction, and positive affect, supported continued engagement. Enjoyment and emotional responses to physical activity also influenced participation. Participants were more likely to continue engaging in activities that were perceived as enjoyable, socially engaging, or meaningful [18]. For example, one participant described how group activities helped them remain active while also providing opportunities for social interaction. The absence of enjoyment or dislike of organized physical activity decreased the motivation level. Organized physical activity involving supervision and social interaction were most likely related to positive experiences. Overview of key results In general, physical activity engagement in older South Asian persons is influenced by a complex interaction of physical capacity, knowledge and belief, environmental and social circumstances, and motivation. Facilitators were most often identified as being associated with social support, safe and supervised environment, and perceptions of good health, while barriers were most often identified as being associated with physical capacity limitations, social roles, and a lack of knowledge regarding physical activity.

## Discussion

This systematic review combines the existing evidence in the field in terms of barriers and facilitators for adopting and adhering to PA in older South Asians in a theoretically informed systematic review fashion. Through the process of presenting the results in the COM-B Model and the TDF, this systematic review has been able to provide a systematic account for the behavioural factors determining PA in older age in the South Asian community through the interrelation of physical constraints, cultural factors, environment, and motivation.

### Interpretation of key findings

Through the studies included in this paper, physical ability has been identified as an overarching factor working against participation in PA. An important finding of this review is that barriers to physical activity among older South Asians often arise from the interaction between ageing-related physical limitations and culturally shaped social roles. While issues such as pain, reduced mobility, and fear of injury are common barriers among older adults generally, the included studies suggest that these physical challenges frequently interact with family responsibilities, caregiving expectations, and cultural beliefs about appropriate behaviour in later life. For example, older adults who experienced physical limitations were sometimes less likely to prioritise physical activity when family or domestic responsibilities were perceived as more important. Perceived physical restrictions owing to ageing factors, ill health, pain, and the risk of accident or injury are common reasons not to participate. Perceived physical limitations related to ageing, illness, pain, and reduced mobility were frequently reported as barriers to physical activity among older South Asians [20, 26, 33]. Participants often described declining strength, fatigue, or fear of injury as limiting their ability to participate in exercise. For example, one participant explained that although walking had been recommended by their doctor, physical frailty made it difficult to follow this advice: “The doctor is saying to walk, but if I cannot do it, how can I do it?” [20]. This has been echoed in other qualitative literature reviews concerning older South Asian populations and older individuals in general, wherein ill health and reduced physical ability are key reasons that influence the desire to participate in PA [8, 9]. A crucial factor here is that older people tend to consider these physical restrictions the natural course of aging.

Limitations in psychological ability, especially the lack of knowledge about what constitutes proper types of PA in old age, were additional factors that limited participation. Misinformation about what constituted valid PA as well as the amounts recommended were at play. A lack of knowledge about PA has been seen in previous studies conducted on South Asians in the past, in which their ordinary daily activities were seen as adequate exercise [7, 8].

Those related to physical opportunity were also found to have an impact. Environmental constraints like costs, climate, lack of facilities, and other home obligations, like taking care of family members, acted as constraining factors for PA participation. Environmental and logistical factors also influenced participation in physical activity. Several studies reported that lack of access to suitable facilities, transportation challenges, and limited space at home acted as barriers to engaging in regular activity [26, 31]. In some cases, participants described living conditions that made exercise difficult. For example, one participant noted: “We cannot do exercise at home. I have a very small place… there is no exercise machine we can keep.” [31]. Facilitating factors, on the other hand, included having access to pathways, free or reduced-cost programs, and supervised sessions. These points were supported by previously conducted reviews, which emphasize the promoting role of environmental accessibility and availability when considering South Asian physical activity participation [10, 11].

Social opportunity was also found to be one of the most stable factors facilitating PA. Group-based activities and support from peers and family members increased motivation and adherence, and lack of family support and lack of fit with family and community expectations, especially among older women, emerged as barriers. The acceptability and effectiveness of socially based exercise and physical activity programs are consistently recognised in a South Asian context and are reflective of a collectivist society where bonding and group interactions are equally important and valued [8, 9]. Social opportunity played an important role in shaping physical activity behaviour. Several studies reported that social norms and family expectations influenced participation in physical activity [31, 33]. In some cases, cultural expectations discouraged women from exercising in public spaces. For example, one participant stated:

“What would the neighbours think if I was walking in the street?” [33].

As far as motivation is concerned, both reflective and automatic processes were involved in PA behaviour. The reflective component was theorised to be driven by beliefs about the benefits to personal health associated with physical activity (PA). These benefits in relation to physical activity included beliefs related to the preservation of independence, prevention, and enhancement of mental health. The automatic component was posited to be driven by experiences related to the affect associated with physical activity. This included enjoyable, social, and supervised experiences related to the activity.

Importantly, these barriers and facilitators did not operate independently. Rather, the synthesis suggests that ageing-related physical limitations interacted with culturally patterned social roles, family expectations, and beliefs about the appropriateness of exercise in later life. For example, pain, fatigue, or fear of injury could reduce not only physical capability but also confidence and motivation, while caregiving responsibilities and family obligations constrained opportunities to engage in activity even where motivation was present. These interacting influences may be especially salient for older South Asians, for whom collectivist family structures, gendered expectations, and culturally shaped understandings of health behaviour can influence whether physical activity is viewed as appropriate, prioritised, or feasible.

Although some barriers identified in this review, such as pain, comorbidity, and fear of injury, are common across older adult populations, the synthesis suggests that among older South Asians these are often embedded within additional cultural and social considerations, including modesty, family duties, community expectations, migration-related adjustment, and preferences for familiar or socially meaningful activity contexts. Cultural norms and social expectations appeared to influence how physical activity was perceived and prioritised among older South Asians. In several studies, participants described concerns about social judgement or cultural expectations surrounding appropriate behaviour in public spaces. These influences may be particularly relevant for older women, whose opportunities for physical activity may be shaped by gender roles, family expectations, and cultural norms. Such findings suggest that culturally sensitive approaches to promoting physical activity may be necessary to ensure that interventions are acceptable and accessible for this population. This helps explain why socially acceptable, group-based, supervised, and culturally familiar forms of physical activity may be particularly important in this population.

### Implications for intervention design

Through the use of the COM-B model and the TDF, this literature review offers specific implications for the design of physical activity interventions among older South Asians. These should include building capabilities through the adaptation of activity to accommodate physical limitations and the provision of reassurance about physical activity and safety. Opportunities should be encouraged through the availability and acceptability of physical activity programmes, taking into consideration cultural and societal factors such as gender and religious views, as well as the inclusion of caregivers. The improvement of motivation should target the promotion of the short-term benefits of physical activity.

It has been suggested that PA interventions, which have a social support component and a group-based focus, conducted in familiar community environments under the guidance of trusted individuals, may be extremely beneficial. The role of health care professionals in encouraging PA has also been important, as health provider advice emerged as a facilitator in a number of studies.

### Relevance to frailty and ageing

Although only one of the studies examined directly measured frailty, a number of study participants reported physical limitations, co-morbidities, and age-related factors which would predispose a participant to frailty. The definition of frailty has been presented as a clinical phenotype [12] and as a building of deficits over time [13]. The barriers and facilitators which emerged from this review are extremely relevant for older South Asians who are frail or at risk of being frail. The findings suggest that the barriers may be compounded by additional cultural and social factors in South Asian communities, such as family responsibilities, community expectations, and migration-related experiences. This highlights the importance of considering both ageing-related and cultural determinants when designing interventions to promote physical activity among older South Asian adults.

### Strengths and limitations

One of the major strengths of this review is that it is theory-driven, which helped to identify the behavioural determinants in an orderly manner. Also, the fact that this is a systematic review, where PRISMA 2020 guidelines are followed for reporting, further increases the reliability of this study.

Despite this, there are several limitations to be considered. Firstly, the relative rarity of qualitative research being specifically conducted among older South Asians restricts the generalisability of the findings among this particular subgroup. Secondly, most included studies were conducted in high-income migrant contexts, particularly in the United Kingdom and Canada. The barriers and facilitators identified may therefore reflect migration-related experiences, local service structures, transport, language, and culturally specific adaptation to host-country settings, and may not fully transfer to South Asian populations living in low- and middle-income settings. Finally, only English-language texts were considered. Conclusion: This qualitative systematic review on older South Asians has shown that engagement in physical activity is influenced by interdependent physical, psychological, social, cultural, and environmental factors. Using the COM-B framework and TDF principles emphasises that capability weaknesses, increasing social and physical facilitators, and raising motivation through enjoyable and meaningful activity participation are important. The findings provide a useful theory-informed basis for developing culturally and age-appropriate physical activity interventions, particularly in high-income migrant settings.

## Supplementary Information

Supplementary Material 1.

Supplementary Material 2.

## Data Availability

All data generated or analysed during this study are included in this published article and its supplementary materials.

## Abbreviations

COM-B: Capability, Opportunity, Motivation–Behaviour
TDF: Theoretical Domains Framework
PA: Physical activity
PRISMA: Preferred Reporting Items for Systematic Reviews and Meta-Analyses
