# Supplementary material for: Barriers and facilitators to physical activity uptake and adherence among older South Asians: a qualitative systematic review
Source: BMC Geriatr. 2026 Apr 18;26:774. doi: 10.1186/s12877-026-07453-3 (PMC13224551; doi:10.1186/s12877-026-07453-3)
Supplement: Supplementary file 1 — Supplementary Material 1. [file 12877_2026_7453_MOESM1_ESM.docx]

**Supplementary File 1**

**Search Strategy**

**Example search strategy for MEDLINE (via Web of Science platform)**

Database: **MEDLINE**
Date searched: **April 2025**
Limits: **English language**

| **Step** | **Search query** |
| --- | --- |
| 1 | “South Asia*” OR India* OR Pakistan* OR Bangladesh* OR Nepal* OR Bhutan* OR “Sri Lanka*” OR Maldiv* |
| 2 | Punjabi OR Bengali OR Urdu OR Tamil |
| 3 | Sikh OR Muslim OR Hindu |
| 4 | diaspora OR immigrant* OR ethnic* |
| 5 | 1 OR 2 OR 3 OR 4 |
| 6 | “physical activity” OR exercise OR walking OR sport OR “active lifestyle” |
| 7 | barrier* OR facilitator* OR perception* OR belief* OR attitude* OR motivation OR participation |
| 8 | qualitative OR interview* OR “focus group*” OR ethnograph* OR thematic OR “content analysis” |
| 9 | 6 AND 7 AND 8 |
| 10 | 5 AND 9 |

The final search combined South Asian population terms with physical activity and qualitative determinants of behaviour.

**Databases searched**

The following databases were searched from **database inception to April 2025**:

- MEDLINE
- Web of Science Core Collection
- CINAHL
- SPORTDiscus
- APA PsycINFO
- Allied and Complementary Medicine Database (AMED)

Search strategies were adapted for each database using database-specific indexing terms and syntax.

**Additional search procedures**

To ensure comprehensive coverage of the literature:

- Reference lists of included studies were screened.
- Relevant systematic reviews were examined to identify additional studies.
- Where study eligibility was unclear, corresponding authors were contacted for clarification.
