# Supplementary material for: Barriers and facilitators to physical activity uptake and adherence among older South Asians: a qualitative systematic review
Source: BMC Geriatr. 2026 Apr 18;26:774. doi: 10.1186/s12877-026-07453-3 (PMC13224551; doi:10.1186/s12877-026-07453-3)
Supplement: Supplementary file 2 — Supplementary Material 2. [file 12877_2026_7453_MOESM2_ESM.docx]

| **Section and Topic** | **Item No.** | **Checklist Item** | **Location where item is reported** |
| --- | --- | --- | --- |
| **TITLE** | 1 | Identify the report as a systematic review | Title page, p. 1 |
| **ABSTRACT** | 2 | See the PRISMA 2020 for Abstracts checklist | Abstract, p. 1 |
| **INTRODUCTION** |  |  |  |
| Rationale | 3 | Describe the rationale for the review in the context of existing knowledge | Introduction, pp. 2–4 |
| Objectives | 4 | Provide an explicit statement of the objective(s) or question(s) the review addresses | Introduction; Aim and Objectives, pp. 5-6 |
| **METHODS** |  |  |  |
| Eligibility criteria | 5 | Specify the inclusion and exclusion criteria for the review and how studies were grouped for synthesis | Methods – Eligibility criteria, pp. 6-7 |
| Information sources | 6 | Specify all databases, registers, websites, organisations, reference lists and other sources searched | Methods – Information sources and search strategy, pp. 6–7 |
| Search strategy | 7 | Present the full search strategies for all databases, including filters and limits | Methods – Information sources and search strategy, pp. 6–7; Supplementary file |
| Selection process | 8 | Specify the methods used to decide whether a study met the inclusion criteria | Methods – Study selection, p. 7 |
| Data collection process | 9 | Specify the methods used to collect data from reports | Methods – Data extraction, p. 8 |
| Data items | 10a | List and define all outcomes for which data were sought | Methods – Data extraction, p. 8 |
|  | 10b | List and define all other variables for which data were sought | Methods – Data extraction, p. 8 |
| Study risk of bias assessment | 11 | Specify the methods used to assess risk of bias in included studies | Methods – Quality appraisal, pp. 8–9 |
| Effect measures | 12 | Specify effect measures used for each outcome | Not applicable (qualitative systematic review) |
| Synthesis methods | 13a | Describe the processes used to decide which studies were eligible for synthesis | Methods – Data synthesis, pp. 8–9 |
|  | 13b | Describe methods required to prepare data for synthesis | Methods – Data synthesis, pp. 8–9 |
|  | 13c | Describe methods used to synthesise results | Methods – Data synthesis (TDF and COM-B mapping), pp. 8–9 |
|  | 13d | Describe any methods used to synthesise results statistically | Not applicable (qualitative systematic review) |
|  | 13e | Describe any methods used to explore possible causes of heterogeneity | Not applicable (qualitative systematic review) |
|  | 13f | Describe any sensitivity analyses conducted | Not applicable (qualitative systematic review) |
| Reporting bias assessment | 14 | Describe any methods used to assess risk of bias due to missing results | Not applicable (qualitative systematic review) |
| Certainty assessment | 15 | Describe any methods used to assess certainty in the body of evidence | Not applicable (qualitative systematic review) |
| **RESULTS** |  |  |  |
| Study selection | 16a | Describe the results of the search and selection process | Results – Study Selection, pp. 10–11; Figure 1 |
|  | 16b | Cite studies that were excluded and explain why | Results – Study Selection, pp. 10–11 |
| Study characteristics | 17 | Cite each included study and present its characteristics | Results – Study Characteristics, pp. 11–14; Table 1 |
| Risk of bias in studies | 18 | Present assessments of risk of bias for each included study | Results – Methodological quality, pp. 14–16; Table 2 |
| Results of individual studies | 19 | Present results for each included study | Results – Construction of barriers and facilitators, pp. 16–22; Table 3 |
| Results of syntheses | 20a | Summarise the characteristics and risk of bias among studies contributing to each synthesis | Results, pp. 11–22 |
|  | 20b | Present results of all statistical syntheses | Not applicable (qualitative systematic review) |
|  | 20c | Present results of investigations of heterogeneity | Not applicable (qualitative systematic review) |
|  | 20d | Present results of sensitivity analyses | Not applicable (qualitative systematic review) |
| Reporting biases | 21 | Present assessments of risk of bias due to missing results | Not applicable (qualitative systematic review) |
| Certainty of evidence | 22 | Present assessments of certainty in the body of evidence | Not applicable (qualitative systematic review) |
| **DISCUSSION** |  |  |  |
| Discussion | 23a | Provide a general interpretation of the results | Discussion, pp. 23–26 |
|  | 23b | Discuss limitations of the evidence included in the review | Discussion – Strengths and Limitations, pp. 26–27 |
|  | 23c | Discuss limitations of the review processes | Discussion – Strengths and Limitations, pp. 26–27 |
|  | 23d | Discuss implications of the results for practice, policy, and future research | Discussion – Implications for intervention design, pp. 25–26 |
| **OTHER INFORMATION** |  |  |  |
| Registration and protocol | 24a | Provide registration information for the review | Methods – Registration protocol (PROSPERO statement), p. 10 |
|  | 24b | Indicate where the review protocol can be accessed | Not applicable |
| Support | 25 | Describe sources of financial or non-financial support | Declarations – Funding, p. 28 |
| Competing interests | 26 | Declare any competing interests | Declarations – Competing interests, p. 28 |
| Availability of data | 27 | Report whether data and materials are publicly available | Declarations – Availability of data and materials, p. 29-30 |
